# Lipocalin 2 expression is associated with aggressive features of endometrial cancer

**DOI:** 10.1186/1471-2407-12-169

**Published:** 2012-05-06

**Authors:** Monica Mannelqvist, Ingunn M Stefansson, Elisabeth Wik, Kanthida Kusonmano, Maria B Raeder, Anne M Øyan, Karl-Henning Kalland, Marsha A Moses, Helga B Salvesen, Lars A Akslen

**Affiliations:** 1 The Gade Institute, Section for Pathology, University of Bergen, Bergen, Norway; 2 Department of Pathology, Haukeland University Hospital, Bergen, Norway; 3 Department of Clinical Medicine, University of Bergen, Bergen, Norway; 4 Department of Gynecology and Obstetrics, Haukeland University Hospital, Bergen, Norway; 5 Computational Biology Unit, Uni Computing, Uni Research AS, Bergen, Norway; 6 Department of Microbiology, Haukeland University Hospital, Bergen, Norway; 7 Vascular Biology Program, Children’s Hospital Boston, Harvard Medical School, Boston, MA, USA; 8 Department of Surgery, Harvard Medical School, Boston, MA, USA

## Abstract

**Background:**

Increased expression of lipocalin 2 (LCN2) has been observed in several cancers. The aim of the present study was to investigate LCN2 in endometrial cancer in relation to clinico-pathologic phenotype, angiogenesis, markers of epithelial-mesenchymal transition (EMT), and patient survival.

**Methods:**

Immunohistochemical staining was performed using a human LCN2 antibody on a population-based series of endometrial cancer patients collected in Hordaland County (Norway) during 1981-1990 (n = 256). Patients were followed from the time of primary surgery until death or last follow-up in 2007. The median follow-up time for survivors was 17 years. Gene expression data from a prospectively collected endometrial cancer series (n = 76) and a publicly available endometrial cancer series (n = 111) was used for gene correlation studies.

**Results:**

Expression of LCN2 protein, found in 49% of the cases, was associated with non-endometrioid histologic type (p = 0.001), nuclear grade 3 (p = 0.001), >50% solid tumor growth (p = 0.001), ER and PR negativity (p = 0.028 and 0.006), and positive EZH2 expression (p < 0.001). LCN2 expression was significantly associated with expression of VEGF-A (p = 0.021), although not with other angiogenesis markers examined (vascular proliferation index, glomeruloid microvascular proliferation, VEGF-C, VEGF-D or bFGF2 expression). Further, LCN2 was not associated with several EMT-related markers (E-cadherin, N-cadherin, P-cadherin, β-catenin), nor with vascular invasion (tumor cells invading lymphatic or blood vessels). Notably, LCN2 was significantly associated with distant tumor recurrences, as well as with the S100A family of metastasis related genes. Patients with tumors showing no LCN2 expression had the best outcome with 81% 5-year survival, compared to 73% for intermediate and 38% for the small subgroup with strong LCN2 staining (p = 0.007). In multivariate analysis, LCN2 expression was an independent prognostic factor in addition to histologic grade and FIGO stage.

**Conclusion:**

Increased LCN2 expression is associated with aggressive features and poor prognosis in endometrial cancer.

## Background

Lipocalin 2 (LCN2), or NGAL, is a secreted glycoprotein belonging to the lipocalin protein family and was first identified as a gene upregulated in mouse kidney cells infected by SV-40 tumor virus [1]. Members of the lipocalin family bind small molecules and cell surface receptors to form macromolecular complexes. They have been previously classified as transport proteins, but it is now clear that they are also involved in several processes related to malignant tumors like cell proliferation, apoptosis and inflammation [2-5].

LCN2 protein is known to be secreted by epithelial cells, macrophages, neutrophils and tumor cells [6,7], and increased levels have been observed in plasma, serum and urine in various conditions such as metastatic breast and colorectal cancer, acute kidney injury, pancreatitis and preeclampsia [8-13]. In tumor tissue, increased expression of LCN2 has been found in human breast, colorectal, ovarian and pancreatic cancers [13-16]. In a mouse model of breast cancer, LCN2 protein expression increased during tumor progression and returned to normal following regression [17].

In malignant tumors, studies have indicated that LCN2 may be involved in epithelial-mesenchymal transition (EMT). Colon carcinoma cells with high LCN2 expression were observed to have decreased cell-cell adhesion due to a dissociation of β-catenin from E-cadherin [15]. Further, E-cadherin expression was down-regulated in breast cancer cell lines overexpressing LCN2 [13], and tumor cells showed an increased motility and invasiveness accompanied by upregulation of mesenchymal markers [13]. In other studies, ovarian cancer cell lines undergoing EMT showed a decreased expression of both LCN2 and E-cadherin [18]. With respect to angiogenesis, studies of pancreatic cancer cells showed LCN2 to block HUVEC endothelial cells tube formation and reduce VEGF secretion [19]. LCN2 has been shown to inhibit tumor angiogenesis by suppressing RAS-induced VEGF expression in 4 T1 tumor cells [20], but to increase angiogenesis in a different breast cancer model [21]. Thus, the interactions between LCN2 and EMT as well as angiogenesis seem to be complex and may be a function of tissue context, tumor type and tumor model.

Recent studies of endometrial cancer have implicated LCN2 in tumor progression. A microarray study showed LCN2 to be the gene with largest fold change between carcinomas and benign tissues such as hyperplasia and normal endometrium. Validation by immunohistochemistry confirmed the increase of LCN2 expression from atypical endometrial hyperplasia to carcinomas [22]. High expression of LCN2 protein together with its receptor SLC22A17 has been related to poorer prognosis among endometrial cancer patients [23]. LCN2 mRNA levels have been associated with different EMT-related genes in a study of endometrial hyperplasia [24]. In endometrial cancer cell lines, LCN2 seems to trigger cytokine production, IL8 being the highest, and this response has been suggested to improve cell survival functions by preventing apoptosis and increase cell migration [25].

The aim of our present study was to investigate LCN2 expression in endometrial tumors with respect to clinico-pathologic phenotype, angiogenesis, EMT markers, vascular invasion by tumor cells, inflammatory markers and patient survival.

## Methods

### Patient series

All 316 patients diagnosed with endometrial carcinoma in Hordaland County (Norway) during the period 1981-1990 were studied. This endometrial cancer series and the variables histologic type, histologic grade, nuclear grade, solid growth, mitoses, estrogen receptor, progesterone receptor, HER-2 expression and FIGO stage, have previously been reported [26-29]. Several markers related to the EMT process (E-cadherin, N-cadherin, P-cadherin, β-catenin) and tumor vascular interactions (VEGF-A, VEGF-C, VEGF-D, bFGF2, blood vascular invasion, lymphatic vascular invasion, vascular proliferation index, glomeruloid microvascular proliferation) have previously been reported and were included for comparison in the present study [27,28,30-32].

Follow-up information was collected from the medical records and correspondence with primary physicians. Patients were followed from the time of primary surgery until death or last follow-up in 2007. The median follow-up time for the survivors was 17 years (range 6 – 23 years); 256 cases with tissue available were included in the current study.

This research was approved by the Norwegian Data Inspectorate (961478–2), Norwegian Social Sciences Data Services (15501), and local ethics committee (REKIII nr. 052.01). Written informed consent for participation in the study was obtained from participants.

### Immunohistochemistry

Staining of LCN2 was performed on 5 μm sections of formalin-fixed and paraffin embedded tumors using tissue microarray (TMA) slides. Sections were boiled for 10 minutes at 750 W followed by 350 W for 15 minutes in 10 mM citrate buffer and stained with a rat monoclonal LCN2 antibody (Clone #220310, MAB1757; R&D Systems, Minneapolis, MN, USA). Pre-treatment with goat serum diluted 1:4 was conducted before incubation with antibody diluted 1:25 for 1 hour at room temperature (RT) followed by 1:300 diluted goat anti-rat IgG-HRP (Santa Cruz, CA, USA) for 1 hour at RT. The peroxidase was localized with diaminobenzidine peroxidase (DAB, Dako, GLostrup, Denmark) as substrate, and sections were counterstained with Dako REAL hematoxylin (Dako).

TMA-slides were evaluated in a standard light microscope (by MM and IMS). Regarding LCN2 expression, cytoplasmic staining intensity in tumor cells (graded 0-3) and staining area (0, no tumor cells positive; 1, <10%; 2, 10%-50%; 3, >50%) were recorded. A staining index (SI) was calculated as a product of staining intensity and positive area giving a staining index of 0-9 [33]. Cases were divided in two subgroups based on the median value (positive cases with SI 1-9 versus negative cases with SI 0). In the survival analysis, the subgroup with strong expression (staining index 9) was shown in addition.

### Gene expression analysis

During 2001-2003, 76 cases of endometrial cancer were prospectively collected at the Department of Gynecology and Obstetrics, Haukeland University Hospital, University of Bergen, Norway. Fresh tumor tissue was carefully dissected from the surgical specimens and was immediately frozen in liquid nitrogen and stored for later use at -80^o^C. Content of tumor cells (by estimated area) was at least 50%, and for the majority >80%.

RNA was extracted with RNeasy Mini Kit (Qiagen, Hilden, Germany) and hybridized to Agilent Whole Human Genome Microarrays 44 k (Cat.no. G4112F), according to the manufacturer’s instructions ( http://www.agilent.com). Arrays were scanned using the Agilent Microarray Scanner Bundle. Microarray signal intensities were determined using BRB-ArrayTools ( http://linus.nci.nih.gov/BRB-ArrayTools/). Mean spot signal data were used as intensity measure. The expression data were normalized, using median over the entire array. This data have been deposited in the ArrayExpress Archive database, http://www.ebi.ac.uk/arrayexpress/ (ArrayExpress accession: E-MTAB-1007).

In addition, a publicly available data set with information on gene expression from endometrial cancer patients was used for supplementary studies (NCBI GEO: GSE2109). The GEO data sets were based on Affymetrix U133 + 2 arrays. For these data sets, individual probes were sequence-matched against Aceview (NCB135) to construct transcript level probe sets [34].

### Statistical methods

Statistical analyses were performed by the PASW statistical software package version 17 (SPSS Inc., Chicago, IL). Associations between different categorical variables were assessed by Pearson’s chi-square test. An association was considered significant if a p-value of <0.05 was obtained. Univariate survival analyses were performed using the Kaplan-Meier method (log-rank significance test). LCN2, together with standard clinico-pathological variables, was further analyzed by log-log plot to determine how these variables could be incorporated in the Cox’ proportional hazards regression model (likelihood ratio significance test).

The PANTHER expression analysis tool was used for gene ontology analysis of microarray data. Gene lists were mapped to PANTHER for categorization of genes to molecular function and biological processes, as well as to biological pathways ( http://pantherdb.org).

## Results

**Figure 1:**
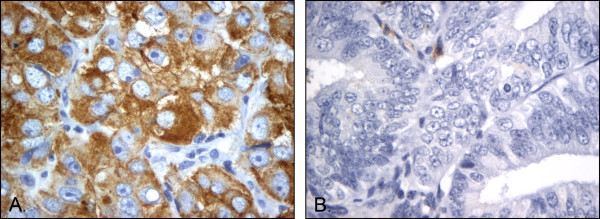
LCN2 protein expression: Immunohistochemical staining showing A) strong and B) no expression of LCN2 in endometrial cancer (magnification x 400).

In total, 125 (49%) of the endometrial cancers were positive for LCN2 cytoplasmic protein expression, while 131 (51%) of the cases were negative (median SI value was 0). Eight endometrial cancers (3%) had a very strong cytoplasmic expression of LCN2 (SI = 9) (Figure 1).

### Clinico-pathologic features

LCN2 protein expression showed a significant association with aggressive features such as non-endometrioid histologic type, high nuclear grade, and predominant solid tumor growth (>50%) (Table 1), but not with FIGO stage or histological grade.

**Table 1:** LCN2 protein expression by various clinico-pathological variables and molecular markers among 256 endometrial cancers

**Variable**		**LCN2 SI 0N (%)**	**LCN2 SI 1-9N (%)**	**P-value^a^**
Histologic type	Endometrioid	126 (55%)	103 (45%)	0.001
	Non- endometrioid	5 (19%)	22 (81%)	
Histologic grade	Grade 1 and 2	89 (56%)	71 (44%)	0.066
	Grade 3	42 (44%)	54 (56%)	
Nuclear grade	Grade 1 and 2	101 (59%)	71 (41%)	0.001
	Grade 3	30 (36%)	54 (64%)	
Solid growth	<50%	104 (56%)	82 (44%)	0.013
	≥50%	27 (39%)	43 (61%)	
Mitoses^b^	Low	103 (54%)	89 (46%)	NS
	High	28 (44%)	36 (56%)	
FIGO stage^c,d^	I/II	109 (53%)	97 (47%)	NS
	III/IV	22 (45%)	27 (55%)	
ER^e,f^	Negative	25 (40%)	38 (60%)	0.028
	Positive	103 (56%)	82 (44%)	
PR^g,h^	Negative	28 (38%)	46 (62%)	0.006
	Positive	98 (57%)	74 (43%)	
EZH2^i,j^	Weak	121 (56%)	94 (44%)	<0.001
	Strong	10 (24%)	31 (76%)	
HER2^k,l^	Weak	115 (54%)	100 (46%)	0.084
	Strong	11 (37%)	19 (63%)	
VEGF-A^m,n^	Weak	114 (55%)	94 (45%)	0.021
	Strong	17 (36%)	30 (64%)	

^a^P-value from *χ*^2 ^test. Mitoses: ^b^median used as cut-off point. FIGO stage: ^c^according to 1998 criteria, ^d^missing data in one case. ER: ^e^missing data in 8 cases, ^f^lower quartile used as cut-off point. PR: ^g^missing data in 8 cases, ^h^lower quartile used as cut-off point. EZH2:^i^missing data in 10 cases, ^j^upper quartile used as cut-off point HER2: ^k^missing data in 11 cases, ^l^median used as cut-off point and VEGF-A:^m^missing data in one case, ^n^upper quartile used as cut-off point.

#### Metastatic pattern

Forty-one of 215 patients (16%) showed recurrence of their primary endometrial cancer during the follow-up period. Regarding the site of recurrent tumors, 34% were located in the vagina, 7% in pelvic lymph nodes, 44% represented distant metastases (liver not included), and 15% were metastases to the liver; 174 cases did not show any spread of the disease. LCN2 expression in the primary tumor was significantly associated with pattern of tumor recurrence (p = 0.029; Table 2), and LCN2 staining was especially related to the development of liver metastases (p = 0.008, Fisher exact test; Table 2).

**Table 2:** Associations between LCN2 expression and metastatic spread among 215 endometrial cancers

**Variable**	**Site of tumor recurrence**	**LCN2 SI 0N (%)**	**LCN2 SI 1-9N (%)**	**P-value^a^**
Recurrent disease^b^	No tumor recurrence	96 (55%)	78 (45%)	0.029
	Vaginal cuff	11 (79%)	3 (21%)	
	Pelvic lymph nodes	2 (67%)	1 (33%)	
	Distant metastasis (not liver)	9 (50%)	9 (50%)	
	Liver	0 (0%)	6 (100%)	

^a^P-value from Pearson’s *χ*^2 ^test, ^b^Missing data for 41 patients.

#### Patient survival

**Figure 2:**
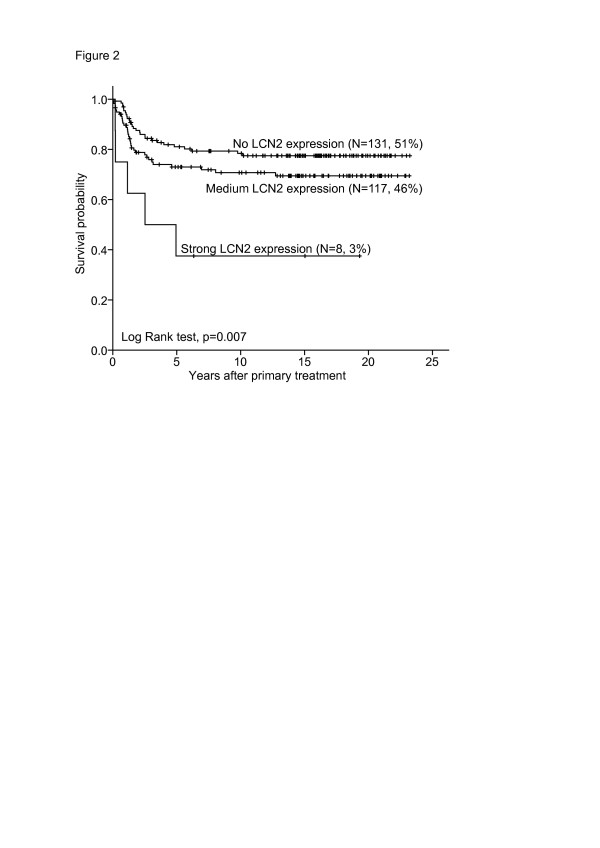
Survival analysis for LCN2: Univariate survival analysis (Kaplan-Meier method, log-rank significance test) for LCN2 in endometrial cancers.

Absence of LCN2 staining was associated with the best survival. Cases with medium staining index (SI 2-6) showed an intermediate survival, whereas the subgroup of patients showing strong LCN2 expression (staining index 9) was associated with the poorest outcome (Figure 2). In multivariate survival analysis, standard clinico-pathologic variables (histologic type, histologic grade, and FIGO stage) were included together with LCN2 expression in three groups. Strong LCN2 was an independent prognostic marker for decreased survival, with Hazard ratio (HR) of 3.9, p = 0.027 (Table 3). Histologic grade (HR 2.8, p < 0.001), and FIGO stage (HR 8.0, p < 0.001) were independent prognostic factors in addition, whereas histologic type was not (HR 1.7, NS) (Table 3).

**Table 3:** Multivariate survival analysis (Cox’ proportional hazards regression model) of clinico-pathologic variables and LCN2 expression in patients with endometrial cancer (n = 255)

**Variables**	**Categories**	**HR^a^**	**95% CI^b^**	**P-value^c^**
LCN2	Negative	1.0		0.027
	Weak/moderate	1.1	0.6-1.9	
	Strong	3.9	1.4-10.8	
Histologic type	Endometrioid	1.0		NS
	Non-endometrioid	1.7	0.9-3.2	
Histologic grade	Grade 1 and 2	1.0		<0.001
	Grade 3	2.8	1.6-4.9	
FIGO stage	I/II	1.0		<0.001
	III/IV	8.0	4.8-13.6	

^a^Adjusted Hazard ratio, ^b^95% confidence interval, ^c^L ratio test.

### Associations with molecular markers

Strong LCN2 expression was significantly associated with ER and PR negative tumors (Table 1), as well as with positive EZH2 expression (p < 0.001). In contrast, LCN2 was not associated with expression of several EMT-related markers such as E-cadherin, N-cadherin, P-cadherin and β-catenin. There was a significant association between LCN2 staining and VEGF-A expression (p = 0.021), whereas LCN2 showed no significant associations with other angiogenesis markers such as vascular proliferation index, glomeruloid microvascular proliferation, VEGF-C, VEGF-D and bFGF2 expression, nor with vascular invasion of tumor cells (in lymphatic or blood vessels).

### Associations with gene expression data

PANTHER expression analysis was used to search for over-represented biological processes and molecular functions among tumors with high (median as cut-point) LCN2 gene expression (fold change >1.5, false discovery rate <10%, p-values <10^-4^) (n = 76). Gene expression among cases with high LCN2 expression was significantly related to integrin signaling as well as the biological processes of angiogenesis and cell adhesion.

Differential gene expression was further examined with respect to known candidate genes for metastatic spread, such as the S100A family in our prospective endometrial cancer series (n = 76) and in the public endometrial cancer series (N = 111) (NCBI GEO: GSE2109). Bivariate correlation analyses of microarray expression data showed a strong and consistent positive correlation between LCN2 gene expression and several S100A-genes, with significant correlations in both series with S100A2, S100A3, S100A6, S100A8, S100A9, S100A11, S100A14 and S100A16 (Table 4).

**Table 4:** Correlation between gene expression for LCN2 and S100-genes in two different microarray data series (Pearson’s correlation)

	**S100A2**	**S100A3**	**S100A6**	**S100A8**	**S100A9**	**S100A11**	**S100A14**	**S100A16**
*In house data series:*								
(n = 76)	0.580	0.271	0.509	0.480	0.480	0.551	0.355	0.340
	<0.001	0.018	<0.001	<0.001	<0.001	<0.001	0.002	0.003
*External data series:*								
(n = 111)	0.509	0.189	0.488	0.346	0.433	0.564	0.423	0.445
	<0.001	0.039	<0.001	<0.001	<0.001	<0.001	<0.001	<0.001

## Discussion

In this study, we demonstrate that LCN2 expression is associated poor outcome in endometrial carcinoma and with aggressive features, including the non-endometrioid histologic type, high grade and solid tumor growth. Similar findings have been reported for other tumors including breast cancer [13-15]. Approximately 50% of the tumors in the present study were positive for LCN2 expression, comparable to breast cancers with staining in 33% of the cases [35].

LCN2 expression has been associated with ER negative, PR negative and HER2 positive breast cancers [13,35-37]. Here, we found an association between LCN2 expression and ER-PR negative endometrial cancers. A similar relationship was observed regarding HER2 status and LCN2, but was of borderline significance only.

Interestingly, LCN2 expression was associated with more distant metastatic spread in our series, especially to the liver, and gene expression data from two independent series supported a relationship between LCN2 and metastasis related genes such as the S100A family [38-42]. In a breast cancer mouse model, plasma samples from preclinical tumor-bearing mice compared with control mice show an upregulated protein expression of S100A8, S100A9, and LCN2 [17]. In addition, S100A8 and S100A9-activated colon cancer cells showed an upregulation of LCN2 gene expression compared to non-stimulated cells [38]. Our findings suggest that LCN2 expression might be implicated in metastases regulation through an interaction with S100A proteins.

Previous studies indicate that LCN2 might be involved in epithelial-mesenchymal transition (EMT) and metastatic spread [13,18,19], consistent with our finding that LCN2 expression is associated with distant metastatic dissemination. Although there were no significant associations with several specific EMT-related protein markers such as cadherins or β-catenin staining, gene expression analysis by PANTHER still indicated a relationship between LCN2 and cell adhesion as well as angiogenesis.

Finally, the data presented here demonstrate that LCN2 expression predicts poor prognosis since cases with strong staining showed a decreased survival compared to those with no staining as demonstrated by multivariate analysis.

## Conclusions

Taken together, our data support associations between LCN2 expression and aggressive tumor features, distant metastatic spread and reduced survival in endometrial cancer. Interestingly, gene expression data indicated a relationship with S100A genes. The mechanism is presently not clear and should be further studied.

## Competing interests

The authors declare that they have no competing interests.

## Authors’ contributions

LAA and MM designed the study with advice from MAM HBS collected prospective tumor samples and provided clinical data. MM, IMS, AMØ, KK, KHK, EW and MBR performed experiments and statistical analyses. All authors participated in the interpretation of results. MM and LAA wrote the manuscript, and IMS, HBS, MAM, AMØ, KHK, EW and MBR commented and edited on the manuscript. All authors read and approved the final manuscript.

## Pre-publication history

The pre-publication history for this paper can be accessed here:

http://www.biomedcentral.com/1471-2407/12/169/prepub
